# Water-Soluble Nanocomposites Containing Co_3_O_4_ Nanoparticles Incorporated in Poly-1-vinyl-1,2,4-triazole

**DOI:** 10.3390/polym15132940

**Published:** 2023-07-04

**Authors:** Artem Emel’yanov, Svetlana Korzhova, Anastasia Ivanova, Tatyana Semenova, Dmitriy Chepenko, Ruslan Usmanov, Alexander Pozdnyakov

**Affiliations:** A.E. Favorsky Irkutsk Institute of Chemistry, Siberian Branch, Russian Academy of Sciences, 1 Favorsky Str., 664033 Irkutsk, Russia; emelyanov@irioch.irk.ru (A.E.); korzhova@irioch.irk.ru (S.K.); ivanova93@irioch.irk.ru (A.I.); semjenova@irioch.irk.ru (T.S.); chepenko@irioch.irk.ru (D.C.); usmanov@irioch.irk.ru (R.U.)

**Keywords:** 1-vinyl-1,2,4-triazole, polymer nanocomposite, cobalt oxide nanoparticles, hydrazine hydrate

## Abstract

New water-soluble nanocomposites with cobalt oxide nanoparticles (Co_3_O_4_NPs) in a poly(1-vinyl-1,2,4-triazole) (PVT) matrix have been synthesized. The PVT used as a stabilizing polymer matrix was obtained by radical polymerization of 1-vinyl-1,2,4-triazole (VT). The polymer nanocomposites with Co_3_O_4_ nanoparticles were characterized by ultraviolet–visible, Fourier-transform infrared spectroscopy, atomic absorption spectroscopy, transmission electron microscopy, dynamic light scattering, gel permeation chromatography, and simultaneous thermogravimetric analysis. The resulting polymer nanocomposites consist of spherical isolated cobalt nanoparticles with a diameter of 1 to 13 nm. The average hydrodynamic diameters of macromolecular coils are 15–112 nm. The cobalt content in nanocomposites ranges from 1.5 to 11.0 wt.%. The thermal stability of nanocomposites is up to 320 °C.

## 1. Introduction

Interest in the study of nanosized particles is associated with the possibility of using nanomaterials in many fields of science and technology, in particular, to obtain efficient and selective catalysts, for example, used in Fischer–Tropsch synthesis [1,2], to create elements of microelectronic and optical devices, and in photocatalysis [3,4] for applications in biology and medicine (biochemical sensors, tumor detection, drug delivery, etc.) [5,6].

Currently, metal nanoparticles stabilized by various polymer matrices are being intensively studied, which makes it possible to combine the unique properties of metal nanoparticles with plasticity, strength, antimicrobial, and other important properties of polymers [7,8]. Magnetic nanocomposite polymeric materials are interesting for study from the point of view of applied and fundamental science [9]. Polymer matrices used as stabilizers improve the properties of nanocomposites: they make it possible to obtain particles with a higher degree of uniformity and a narrow range of particle size distribution, which makes them possible to use in medicine [10,11,12,13].

Among numerous magnetic nanomaterials, cobalt nanoparticles, prepared by different methods, attract special attention because of their electrical, magnetic, and catalytic properties [14]. Spin-dependent electron transport on 10 nm diameter cobalt nanocrystals showed according to C.T. Black et al. [15]. The cobalt wire has potential in applications such as materials for magnetic antennas [16]. Due to the high saturation magnetic induction and high catalytic activity, nanodispersed cobalt is a promising material for the creation of magnetic fluids and permanent magnets, compact composite materials, as catalysts, magnetic sensors, and is also widely used in medicine and biology [17,18]. Spherical cobalt nanoparticles with a diameter of 400 nm were produced by employing the liquid-phase reduction method and hydrazine [19]. Cobalt oxides CoO and Co_3_O_4_ with an average size of 50–100 nm were obtained by thermal decomposition methods from Co (II) acetate tetrahydrate [20]. Monodispersed, stabilized, defect-free ε-cobalt nanocrystals with spherical shapes and sizes ranging from 3 to 17 nm, as well as cubic and rod-like shaped particles were produced by the rapid pyrolysis of cobalt carbonyl in an inert atmosphere [21]. The cobalt-substituted magnetite nanoparticle sizes from 7 to 50 nm with controlled self-assembly and magnetic alignment were produced by Yongsheng Yu et al. [22]. Co^2+^ and Co nanoparticles interconnected triazine-based porous organic polymer proved to be a highly efficient electrocatalyst for the water-splitting reaction [23].

Cobalt oxides also attract attention. Co_3_O_4_ is an inorganic semiconductor and has a wide range of potential applications in the field of heterogeneous catalysis, in magnetic materials and electrochemical devices, and as anode materials for rechargeable lithium-ion batteries. For example, monodispersed Co_3_O_4_ nanocubes with a size of 3–10 nm coated on carbon nanotubes has excellent electrochemical activity and is promising as an anode material for sodium-ion batteries [24]. Nanofibers with Co_3_O_4_ nanoparticles with an average diameter of 52 nm can be used for the fabrication of exhaled breath sensors in pulmonary disease detection [25]. It has low toxicity, high theoretical specific capacity, and good cycling life, as well as excellent redox ability [26,27,28,29]. To achieve better performance of devices based on such materials, it is desirable to develop inorganic materials with controlled sizes, shapes, and structures. Obtaining stable colloids of cobalt nanoparticles is hampered by their tendency to oxidize in air and strong magnetic interaction between nanoparticles. Therefore, it is necessary to use a polymer matrix as a stabilizer of nanoparticles to control their growth and prevent oxidation [30].

Poly-1-vinyl-1,2,4-triazole (PVT) was used as a polymer matrix, which is highly soluble in water and resistant to acid and alkaline hydrolysis. Additionally, PVT is non-toxic, which has been established on outbred white mice. The lethal dose (LD_50_), at single and intragastric injection of PVT, exceeds 5000 mg/kg [31,32,33]. Previously, we found that PVT can act as an effective stabilizing matrix for silver, gold, and iron nanoparticles and provides the resulting nanocomposites with valuable properties: water solubility, antimicrobial activity, chemical stability, and heat resistance [34,35,36].

In this work, the synthesis and characterization of new water-soluble nanocomposites containing Co_3_O_4_ nanoparticles incorporated in non-toxic poly-1-vinyl-1,2,4-triazole is reported. The structure and properties of nanocomposites are shown, as well as the effect of the ratio of polymer and cobalt(II) acetate tetrahydrate on the size of cobalt oxide nanoparticles and their polydispersity.

## 2. Materials and Methods

### 2.1. Materials

The initial monomer 1-vinyl-1,2,4-triazole (VT) was synthesized according to the method in [37]. Azobisisobutyronitrile (AIBN) (CAS Number: 78-67-1, 99%, Sigma-Aldrich, Munich, Germany), cobalt(II) acetate tetrahydrate (Co(CH_3_COO)_2_∙4H_2_O) (CAS Number: 6147-53-1, Sigma-Aldrich, Munich, Germany), and hydrazine hydrate (CAS Number: 10217-52-4, Sigma-Aldrich, Munich, Germany) were used as received without further purification. Dimethylformamide (DMF) turned into acetone was distilled and purified according to the known procedures. H_2_O was used as deionized and degassed.

### 2.2. Synthesis of Poly-1-vinil-1,2,4-triazole (PVT)

**Scheme 1:**
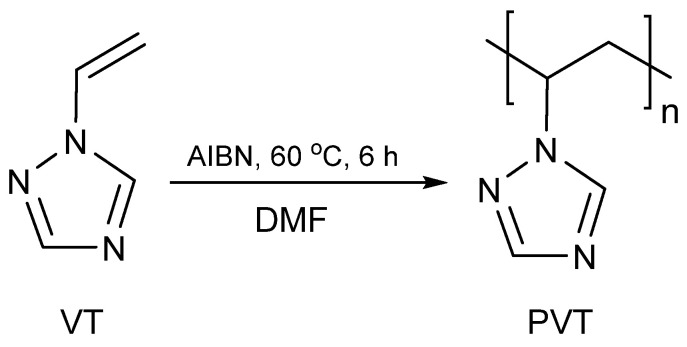
The synthesis of poly(1-vinyl-1,2,4-triazole).

Poly-1-vinyl-1,2,4-triazole was synthesized by radical polymerization of 1-vinyl-1,2,4-triazole in the presence of azobisisobutyronitrile (1%) at 60 °C in DMFA for 6 h. (Scheme 1).

1-Vinil-1,2,4-triazole (1.5 g; 15.8 mmol), AIBN (0.018 g; 0.1 mmol), and DMF (1.0 g) were placed in an ampoule. Then the glass ampoule was filled with argon and sealed. The mixture in the ampoule was stirred and kept in a thermostat at 60 °C for 6 h until the completion of polymerization. A light-yellow transparent block was formed. After the polymerization, ampoules were opened, the reaction mixture was dissolved in DMF and precipitated into acetone. The synthesized PVT was dried in a vacuum oven. Then the polymer was dissolved in water and purified by dialysis for 48 h through a cellulose membrane with a pore size of 5 kDa (Cellu Sep H1, MFPI, Seguin, TX, USA).

### 2.3. Synthesis of Complexes PVT-Co^2+^

PVT-Co^2+^ metal-polymer complexes were obtained by mixing aqueous solutions of poly-1-vinil-1,2,4-triazole (the concentration in water was 2.7∙10^−2^–1.1 mol/L) and cobalt(II) acetate tetrahydrate with a concentration of 2.7∙10^−2^ mol/L with constant stirring for 20 min, varying the ratio of the polymer to Co^2+^ from 5:1 to 40:1.

### 2.4. Synthesis of Nanocomposites PVT-Co_3_O_4_NPs

For the synthesis of cobalt-containing nanocomposites, the method of chemical reduction of cobalt ions to the metallic state from a solution of its complex was used. The molar ratios of polymer:metal ions were 5:1, 10:1, 20:1, and 40:1. Hydrazine hydrate was used as a reducing agent in a tenfold molar excess with respect to Co^2+^, which was added dropwise over 3 min with constant stirring, while observed foaming and color changed over time from sandy to gray-violet. The reaction mixture was stirred for 3 h, precipitated with cold acetone. The precipitate was separated by centrifugation and dried in a vacuum oven to constant weight. The synthesized nanocomposites 1 and 2 are soluble in water.

### 2.5. Characterization Methods

The molecular weight of the polymer was determined by gel permeation chromatography at 50 °C using Shimadzu LC-20 Prominence system (Shimadzu Corporation, Kyoto, Japan) fitted with a differential refractive index detector, Shimadzu RID-20A, and column Agilent PolyPore 7.5 × 300 mm (PL1113-6500). N,N-Dimethylformamide was used as the eluent at the flow rate of 1 mL/min. Dissolution of samples was performed at 50 °C for 24 h with stirring. Calibration was carried out using a series of polystyrene standards, Polystyrene High EasiVials (PL2010-0201), consisting of 12 samples with molecular weights from 162 to 6,570,000 g/mol. The optical spectra of the complexes and nanocomposites were studied on a Shimadzu UV-2450 spectrophotometer with a wavelength from 190 to 900 nm (Shimadzu Corporation, Kyoto, Japan). FTIR spectra were recorded on a Varian 3100 FTIR spectrometer. The cobalt content in the studied nanocomposites was estimated by atomic absorption analysis using a Shimadzu AA-7000 (Shimadzu Corporation, Kyoto, Japan). Microphotographs were obtained using a transmission electron microscope (Leo 906E, Zeiss, Germany). The hydrodynamic particle diameter (D_h_) of the studied copolymers was determined in a water-salt solution (NaNO_3_ 0.1 M) with 0.1 mg·ml^−1^ copolymer concentration; we used the dynamic light scattering (DLS) method on a ZetaPALS zeta potential analyzer with a BI-MAS module (Brookhaven Instruments Corporation, Holtsville, NY, USA). The survey was carried out in polystyrene cuvettes, the survey angle was 90°, and the wavelength was 659 nm. X-ray diffraction (XRD) were obtained on a powder diffractometer (D8 Advance, Bruker Corporation, Germany, Cu radiation). Thermogravimetric analysis (TGA) and differential scanning calorimetry (DSC) were performed using STA 449 Jupiter (Netzsch, Germany) in air atmosphere at a heating rate of 5 °C per min from 30 to 1000 °C; the weight of the samples was 10 mg. Analysis of the qualitative and quantitative composition of the evolved gaseous thermolysis products was performed using a QMS 403 C Aeolos quadrupole mass spectrometer (Netzsch, Selb, Germany) coupled with the thermal analyzer. The surface structure was studied by scanning electron microscopy on a Philips 525-M instrument (Royal Philips Electronics Inc., Amsterdam, The Netherlands). EDS was studied by FEI Quanta 200 scanning electron microscope (FEI Company, Hillsboro, OR, USA) with EDAX X-ray microanalysis attachment with nitrogen-free cooling GENESIS XM 2 60-Imaging SEM with APOLLO 10.

## 3. Results and Discussion

### 3.1. Synthesis of Metal-Polymer Complexes (PVT-Co^2+^)

**Figure 1:**
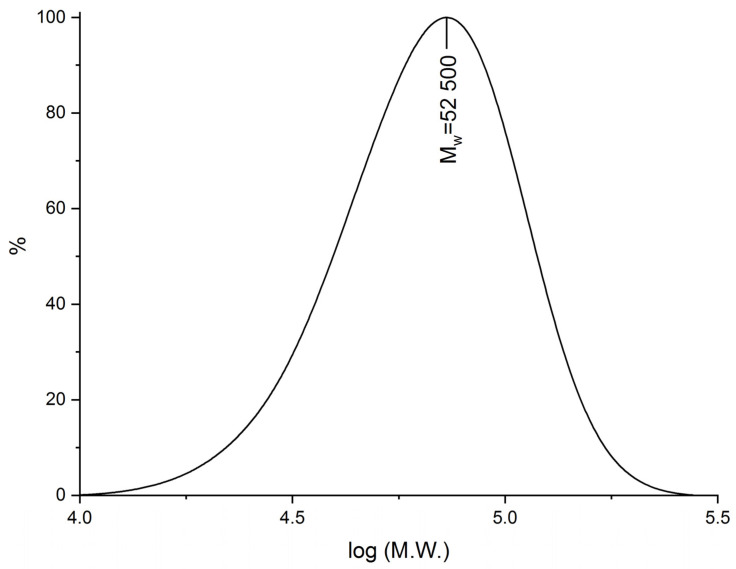
Molecular weight distribution of poly-1-vinyl-1,2,4-triazole.

The PVT was obtained in 93% yield as a white powder with M_w_ 52,500 Da, PDI 2.01 and used for the subsequent synthesis of the metal–polymer complexes and nanocomposites (Figure 1).

The synthesis of nanocomposites with cobalt oxide nanoparticles in a polymer matrix was carried out in two stages. At the first stage, to obtain a metal-polymer complex (PVT-Co^2+^), aqueous solutions of the initial polymer and cobalt(II) acetate tetrahydrate were mixed at various polymer:metal ion molar ratios in the range from 5:1 to 40:1.

**Figure 2:**
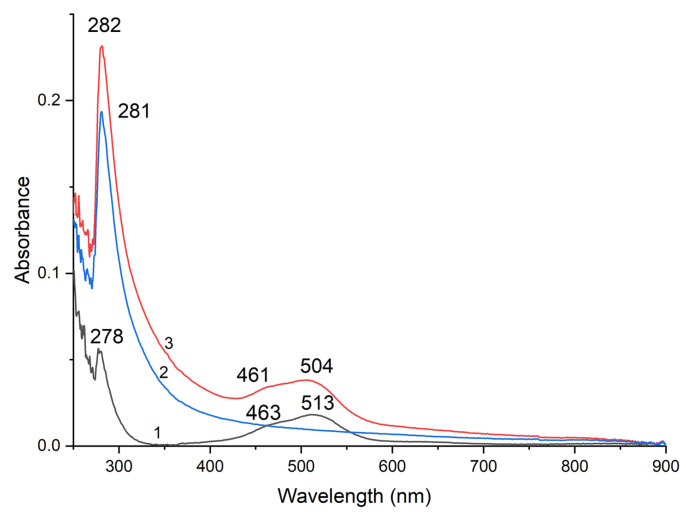
Optical absorption spectra of the initial compounds: 1—Co(CH_3_COO)_2_∙4H_2_O (concentration 2.7∙10^−2^ mol/L), 2—PVT (concentration 2.7∙10^−1^ mol/L), and 3—PVT-Co^2+^ complexes with the molar ratio polymer:metal ion from 10:1.

The formation of complexes was confirmed by ultraviolet–visible (UV) and Fourier-transform infrared spectroscopy (FTIR) spectroscopy data. In the optical absorption spectra of the initial PVT, bands with a maximum λ_max_ 206 nm for the π−σ* transitions of the C=N group of the triazole ring and with λ_max_ 281 nm for the π−π* transition of the same group are observed (Figure 2) [38,39]. The optical absorption spectra of cobalt(II) acetate tetrahydrate contain the bands with a maximum at λ_max_ 205, 278, 463, and 513 nm.

After adding the Co^2+^ salt solution to the initial poly-1-vinyl-1,2,4-triazole solution, the band Co^2+^ salt with a maximum at 278 nm shifts to 282 nm. During the formation of the complex, a shift of the maximum of the absorption band of the initial solution of cobalt(II) acetate tetrahydrate in the visible region with a wavelength of λ_max_ 513 (absorption value 0.018) to a shorter wavelength region up to λ_max_ 505 nm (absorption value 0.038) occurs. The observed hyperchromic effect, as well as a shift in the ultraviolet region, proves the formation of complexes.

**Figure 3:**
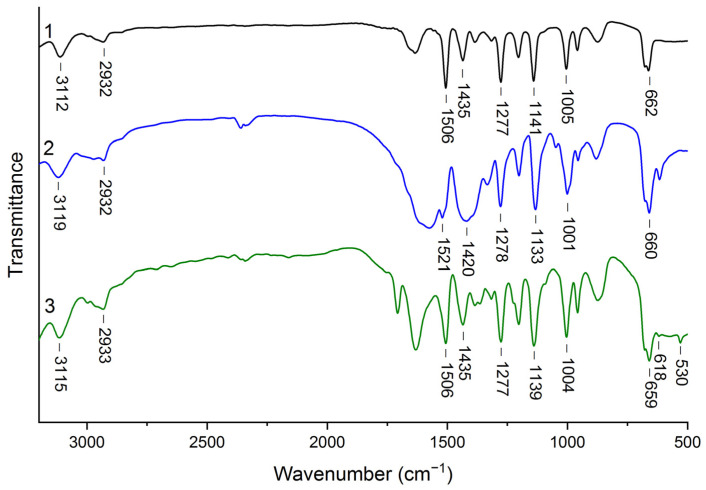
FT-IR spectra: 1—PVT, 2—complex with a polymer:metal molar ratio of 5:1, and 3—nanocomposite with a polymer:metal molar ratio of 20:1.

The FT-IR spectra of the initial poly-1-vinyl-1,2,4-triazole and PVT-Co^2+^ complexes are shown in Figure 3. The FT-IR spectrum of poly-1-vinyl-1,2,4-triazole contains characteristic absorption bands corresponding to the stretching and bending vibrations of the triazole ring at 3112 cm^−1^ (C–H), 1506 cm^−1^ (C=N), 1435 cm^−1^ (C–N), 1277 cm^−1^ (N–N), 1141, 1005 cm^−1^ (C–H), 662 cm^−1^ (C–N), and characteristic absorption bands of the polymer chain at 2932 cm^−1^ (CH, CH_2_).

In the IR spectrum of the resulting PVT-Co^2+^ complex, there is a shift of the absorption band of the C=N bonds of the heterocycle to the low-frequency region by 15 cm^−1^ (1521 cm^−1^) compared to the initial PVT (Figure 3). The spectrum of the complex also shows the shift of the absorption band of the C–N bond to the high-frequency region is 15 cm^−1^ (1420 cm^−1^). The absorption bands of C–H bonds of the triazole ring also undergo changes, shifting to the high-frequency region by 8 cm^−1^ (1133 cm^−1^) and 4 cm^−1^ (1001 cm^−1^) compared to the initial PVT. There is also a shift of the bands of C–H bonds to the low-frequency region by 7 cm^−1^ (3119 cm^−1^). Similar shifts of stretching and bending vibration bands are characteristic of triazole-containing metal complex compounds [40]. All this indicates the coordination interaction of cobalt ions with the pyridine nitrogen atom of the triazole ring, which is characterized by an increased electron density, possesses donor properties, and is able to efficiently enter into coordination interactions with metal ions [41,42], which was shown by nuclear magnetic resonance method [43].

### 3.2. Synthesis of Nanocomposites with Cobalt Oxide Nanoparticles (Co_3_O_4_NPs)

At the second stage, nanocomposites with cobalt oxide nanoparticles were obtained. To obtain Co_3_O_4_NPs, the method of chemical reduction of cobalt ions from a solution of PVT-Co^2+^ complex was used. The reduction was carried out in an aqueous medium with constant stirring in the presence of a reducing agent. Hydrazine hydrate, which is widely used for the synthesis of nanocomposites, was used as a reducing agent [44,45]. Its use ensures the completeness of reduction, fast redox reaction, and the obtaining of pure target products [46].

As a result, cobalt-containing nanocomposites with a cobalt content of 1.5 to 11 wt.%, depending on the initial molar ratio of the stabilizing polymer and metal ion, are obtained (Table 1).

**Figure 4:**
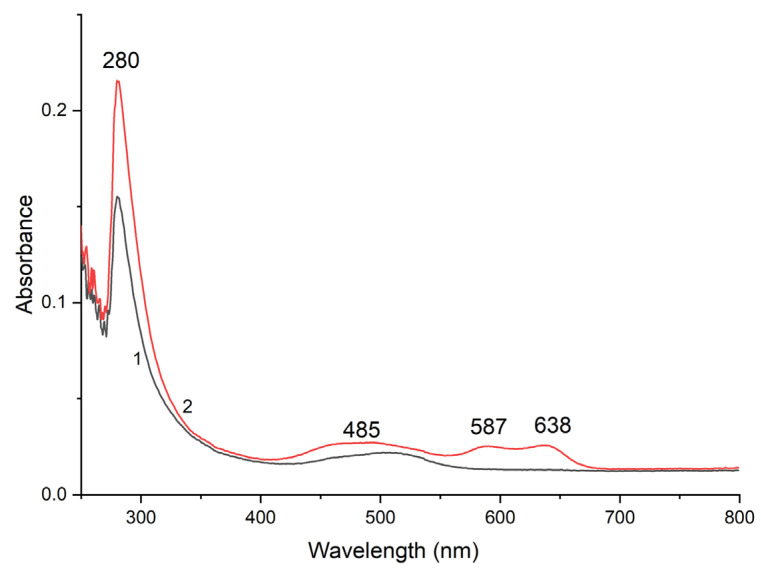
Optical absorption spectra: 1—PVT-Co^2+^ complex and 2—nanocomposite after 1 min from the reaction beginning (molar ratio polymer:metal ion 20:1).

The formation of nanocomposites was monitored using UV spectroscopy. Figure 4 shows that one minute after the addition of hydrazine hydrate, the reduction of cobalt ions begins. The UV–Vis absorption spectra show that the polymer nanocomposites have an absorption peak at 280 nm.

On the optical absorption spectra of the obtained aqueous solutions of nanocomposites with cobalt oxide nanoparticles in a poly-1-vinyl-1,2,4-triazole matrix in the region between 420 and 675 nm, the appearance of absorption bands with maxima at 485, 587, and 638 nm is observed (Figure 4).

The peak with a maximum at 485 nm, characteristic of Co(II) in the octahedral coordination, and a broad absorption doublet with a maximum at 587 nm and 638 nm, characteristic of Co(III) in the tetrahedral coordination, indicate the formation of Co_3_O_4_ [47,48,49].

**Figure 5:**
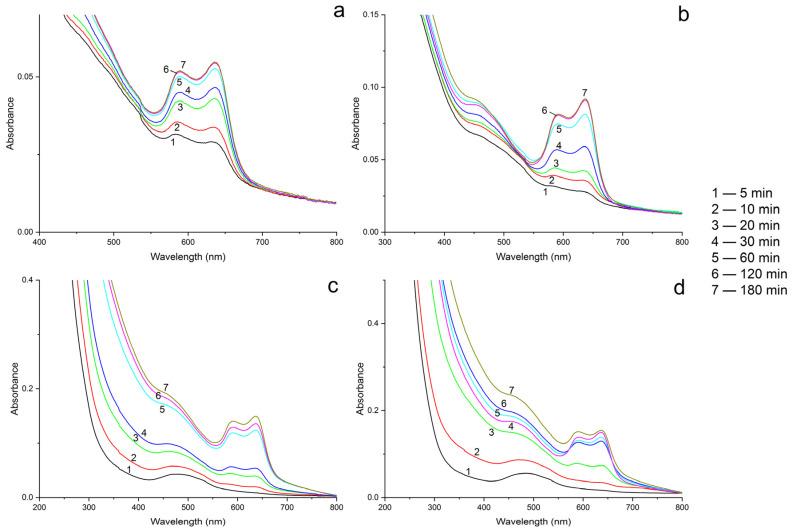
Optical absorption spectra of the resulting nanocomposites with different polymer:metal ion molar ratios: (**a**) 40:1, (**b**) 20:1, (**c**) 10:1, and (**d**) 5:1 from 5 to 180 min.

Figure 5 shows the electronic absorption spectra of nanocomposites 1–4 after adding hydrazine hydrate. Five minutes after the start of the reduction reaction, the formation of cobalt oxide nanoparticles in solution was observed. With a cobalt content of up to 2.8 wt.%, the reduction reaction is completed after 120 min (Figure 5a,b); with a cobalt content of 5 to 11 wt.%—after 180 min (Figure 5c,d). The optical density increases with an increase in the cobalt content in the nanocomposite.

To assess the stabilizing ability of the PVT, centrifugation was performed at 10,000 rpm for 15 min. The plasmon absorption band of cobalt nanoparticles did not change after centrifugation, which indicates a high stabilizing ability of polymer matrix.

An analysis of the IR spectra of the obtained nanocomposites with cobalt oxide nanoparticles in a poly-1-vinyl-1,2,4-triazole matrix shows that there are no significant changes in the polymer matrix (Figure 3). The IR spectrum contains small peaks with maxima at 530 and 618 cm^−1^, which correspond to the presence of cobalt oxide in the nanocomposite [25,47,50,51].

According to atomic absorption spectroscopy, the cobalt content in nanocomposites varies from 1.5 to 11.0 wt.%. The content of cobalt depends on the polymer:metal ion molar ratios. The stabilizing ability of the polymer matrix decreases with an increase in the cobalt content relative to the polymer. This leads to partial coagulation and the formation of larger nanoparticles. An increase in the cobalt content above 5.8 wt.% led to a partial loss of solubility of nanocomposites 3 and 4 in water and dipolar organic solvents.

Aqueous-salt solutions (NaNO_3_ 0.1 M) with 0.1 mg mL^−1^ copolymer concentration of nanocomposites and original PVT were analyzed by dynamic light scattering. The resulting distribution diagrams of scattering particles are characterized by maxima corresponding to the average hydrodynamic diameters of macromolecular coils. The original PVT is characterized by a unimodal peak with a maximum corresponding to macromolecular particles with a hydrodynamic diameter of 9 nm, which are in solution in the form of individual polymer chains. The data obtained are consistent with the molecular weight characteristics of the polymer, which were determined by gel permeation chromatography.

**Figure 6:**
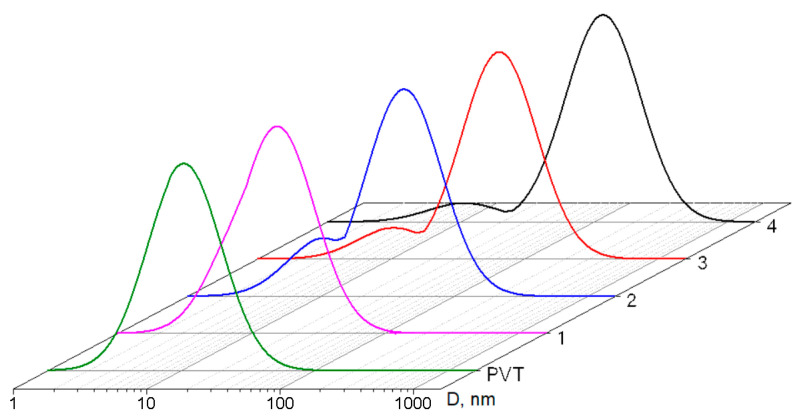
Histogram of the distribution over the hydrodynamic diameters of the scattering particles for the PVT and nanocomposites 1–4.

Histograms of hydrodynamic diameters of nanocomposites in an aqueous-salt solution are characterized by a unimodal (40:1) and bimodal (20:1, 10:1, 5:1) distribution (Figure 6). It has been established that the formation of Co_3_O_4_ nanoparticles in the presence of PVT leads to the formation of a dispersed phase of nanocomposites, the hydrodynamic dimensions of which correlate with the content of cobalt. The introduction of 1.5 wt.% cobalt in the form of Co_3_O_4_ nanoparticles into the polymer matrix leads to an increase in the average hydrodynamic diameter of macromolecular coils of the nanocomposite to 15 nm. A subsequent increase in the content of cobalt oxide nanoparticles to 11.0 wt.% leads to a consistent increase in the average hydrodynamic diameters of polymer nanocomposites up to 112 nm (Table 1). At the same time, in the histogram of nanocomposites 2–4, in addition to the slow mode corresponding to the scattering particles of the nanocomposite, there is a fast mode, which corresponds to the initial PVT. The intensity of the fast mode decreases with increasing cobalt content. This indicates the presence of free polymer chains that are not involved in the stabilization of Co_3_O_4_NPs.

Thus, nanocomposites are high-molecular compounds consisting of Co_3_O_4_NPs in a PVT polymer matrix. Stabilization of Co_3_O_4_ particles formed as a result of reduction processes is ensured by the formation of coordination bonds of triazole rings with cobalt oxide nanoparticles. In this case, the resulting bond of nanoparticles with the PVT matrix will be greatly enhanced by cooperative multipoint coordination binding simultaneously with the whole ensemble of many rigidly interconnected coordination centers located on the surface of the nanoparticles. An increase in the content of Co_3_O_4_NPs in the stabilizing matrix of PVT leads to the formation of three-dimensional cross-linked structures, whose average hydrodynamic diameter increases with an increasing percentage of cobalt. A similar interaction of nanoparticles and a stabilizing polymer matrix was observed in the poly-N-vinylimidazole—copper nanoparticles system [52].

**Figure 7:**
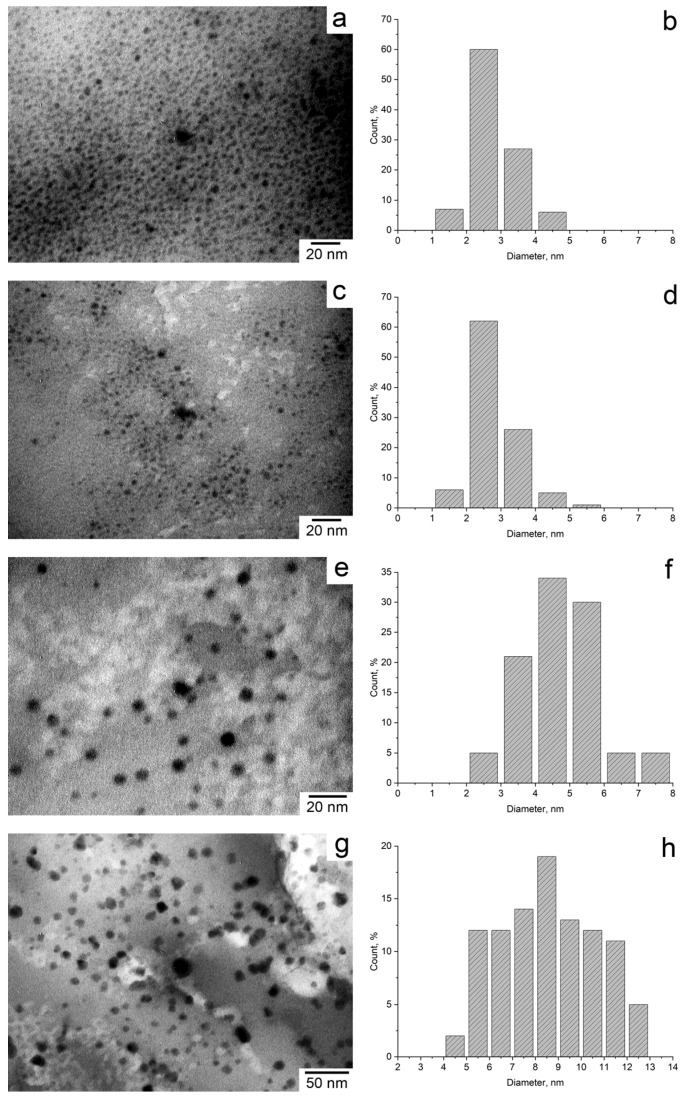
Electron micrographs of nanocomposites 1–4 (**a**,**c**,**e**,**g**) and size distribution diagrams of cobalt oxide nanoparticles (**b**,**d**,**f**,**h**).

The shape and size of nanoparticles in nanocomposites 1–4, as well as their distribution in the polymer matrix, were studied using transmission electron microscopy. The synthesized polymer nanocomposites consist of isolated nanoparticles of cobalt oxides, predominantly spherical in shape, distributed in the bulk of the polymer matrix (Figure 7) with particle sizes of 1–13 nm.

The content of cobalt in nanocomposites 1–4 affects the sizes of nanoparticles. The smallest size distribution of nanoparticles is observed in nanocomposite 2 (Figure 7), in which the cobalt content is minimal, where particles with sizes of 3–4 nm make up 88.5%.

Weight averages (*D_w_*) and number averages (*D_n_*) regarding nanoparticle diameter, and polydispersity indices (PDI) were calculated from nanoparticle size data using the following equations (Table 2) [37]:

$$\begin{array}{l} D_{n}=\frac{\sum_{i}n_{i}D_{i}}{\sum_{i}n_{i}}\\ D_{w}=\frac{\sum_{i}n_{i}D_{i}^{4}}{\sum_{i}n_{i}D_{i}^{3}}\\ PDI=D_{w}/D_{n} \end{array}$$

where *n_i_* is the number of particles of size *D_i_*.

Table data show that cobalt oxide nanoparticles in nanocomposites 1–4 have a narrow size dispersion. An increase in the cobalt content in the nanocomposites from 1.5 to 11.0 wt.% leads to an increase in the nanoparticle sizes from 3.4 to 10.0 nm (*D_w_*) and from 2.7 to 8.6 nm (*D_n_*). The PDI of cobalt oxide nanoparticles in nanocomposites 1–4 decreases from 1.23 to 1.16.

**Figure 8:**
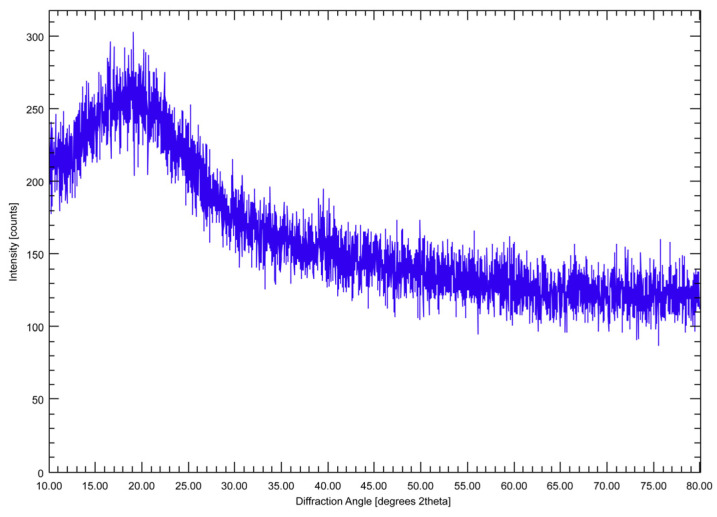
X-ray diffraction pattern for nanocomposite 2.

The diffraction pattern of the nanocomposite showed an amorphous halo of the polymer matrix (Figure 8). The Co nanoparticles obtained by us by the described synthesis method have an amorphous structure. Similar amorphous structures are described in the works by Wu et al. and Markova-Deneva [53,54].

**Figure 9:**
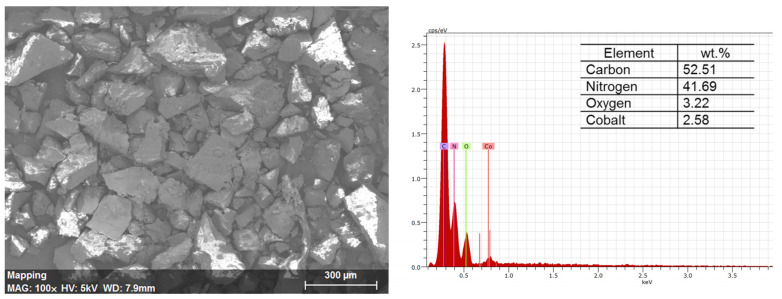
Scanning electron micrograph and energy-dispersive X-ray spectroscopy for the nanocomposite 2.

Scanning electron microscopy data show the dense structure of the nanocomposite. The cobalt content determined by energy dispersive X-ray spectroscopy is 2.6 wt.% (Figure 9).

**Figure 10:**
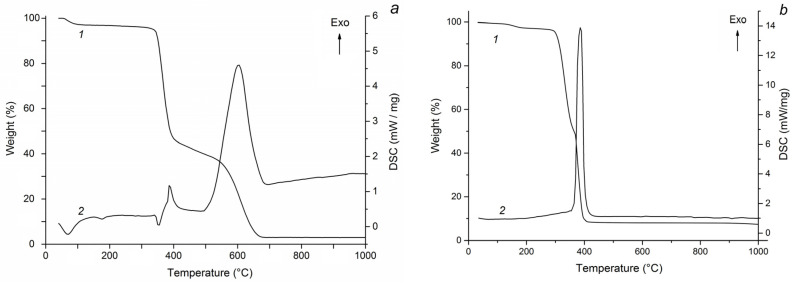
TGA (1) and DSC (2) curves of (**a**) poly-1-vinyl-1,2,4-triazole and (**b**) nanocomposite 2 with cobalt oxide nanoparticles.

The synthesized nanocomposite with cobalt oxide nanoparticles was studied by TGA and DSC methods with mass spectrometry of thermolysis gas products. The thermal stability of nanocomposite 2 according to thermogravimetric analysis is 300 °C. Thermal decomposition of the nanocomposite 2 begins at 60–180 °C, and adsorbed water is released, as evidenced by the appearance in the mass spectrum of a signal with a mass number of 18 with a weight loss of 3.5% (Figure 10).

Further, at a temperature of 280–360 °C, the triazole groups of the polymer are destroyed and oxidized with the release of H_2_O, CO_2_, and NO_2_ (mass numbers 18, 44, 46). A weak exothermic effect is observed with maximum at 360 °C. The weight loss of the sample is 48%.

The last stage of nanocomposite degradation occurs at 370–410 °C. On DSC and TGA curves, an exothermic effect with a maximum at 386 °C is observed at a sample weight loss of 41%. In this case, the carbon skeleton of the main polymer chain is burned out. Decomposition products are oxidized to form CO_2_ (mass number 44).

The temperatures at 10% weight loss (T_10_) and 50% weight loss (T_50_) for the nanocomposite 2 are 211 °C and 367 °C, respectively. The difference between the remaining mass of PVT and nanocomposite 2 is 4.5%, which indicates the presence of Co_3_O_4_NPs. The decrease in the thermal stability of nanocomposites compared to PVT is due to the catalytic features of Co_3_O_4_NPs, which reduce the activation energy of thermal destruction and oxidation of the polymer matrix.

## 4. Conclusions

This manuscript presents data on the synthesis of new water-soluble polymer nanocomposites with cobalt oxide nanoparticles content in poly(1-vinyl-1,2,4-triazole) matrix. The use of PVT provides effective stabilization of cobalt nanoparticles, which is achieved due to the formation of coordination bonds between the surface of nanoparticles and the coordination centers of the polymer ligand. It was found that the ratio of the polymer and cobalt(II) salt in the initial reaction mixture affects the size of cobalt oxide nanoparticles and their polydispersity. An increase in the cobalt content leads to the formation of a nanocomposite with a large size distribution of nanoparticles from 1 to 5 nm to 4 to 13 nm. According to DLS data, the average hydrodynamic diameters of macromolecular coils increase from 15 to 112 nm with an increase in the cobalt content in nanocomposites. New water-soluble nanocomposites containing Co_3_O_4_ nanoparticles in poly-1-vinyl-1,2,4-triazole matrix are promising materials for the design of new components for applications in medicine and biology. In continuation of this work, it is planned to study the antimicrobial activity of the synthesized nanocomposites against pathogenic microorganisms.

## Figures and Tables

**Table 1:** Composition and characteristics of the nanocomposites with Co_3_O_4_NPs 1–4.

Nanocomposite	PVT:Co(II), mol	Yield, %	Co Content, %wt	Nanoparticle Size, nm	Average Hydrodynamic Diameter, nm
1	40:1	82.1	1.5	1–6	15
2	20:1	82.7	2.8	1–6	41
3	10:1	83.4	5.8	2–8	66
4	5:1	84.6	11.0	4–13	112

**Table 2:** Average size (*D_w_*, *D_n_*) and polydispersity of nanoparticles in nanocomposites 1–4.

Nanocomposite	*D_w_*, nm	*D_n_*, nm	PDI
1	3.37	2.72	1.23
2	3.33	2.80	1.19
3	5.79	4.88	1.18
4	10.03	8.62	1.16

## Data Availability

Not applicable.
